# Stability and anisotropy of (Fe_x_Ni_1−x_)_2_O under high pressure and implications in Earth’s and super-Earths’ core

**DOI:** 10.1038/s41598-017-18678-z

**Published:** 2018-01-10

**Authors:** Shengxuan Huang, Xiang Wu, Shan Qin

**Affiliations:** 1 State key laboratory of geological processes and mineral resources, China University of Geosciences (Wuhan), Wuhan, 430074 P. R. China; 2 Key Laboratory of Orogenic Belts and Crustal Evolution, MOE, Peking University and School of Earth and Space Sciences, Peking University, Beijing, 100871 P. R. China

## Abstract

Oxygen is thought to be an important light element in Earth’s core but the amount of oxygen in Earth’s core remains elusive. In addition, iron-rich iron oxides are of great interest and significance in the field of geoscience and condensed matter physics. Here, static calculations based on density functional theory demonstrate that *I*4/*mmm*-Fe_2_O is dynamically and mechanically stable and becomes energetically favorable with respect to the assemblage of *hcp*-Fe and $R\bar{3}m$-FeO above 270 GPa, which indicates that *I*4/*mmm*-Fe_2_O can be a strong candidate phase for stable iron-rich iron oxides at high pressure, perhaps even at high temperature. The elasticity and anisotropy of *I*4/*mmm*-(Fe_x_Ni_1−x_)_2_O at high pressures are also determined. Based on these results, we have derived the upper limit of oxygen to be 4.3 wt% in Earth’s lower outer core. On the other hand, *I*4/*mmm*-(Fe_x_Ni_1−x_)_2_O with high *AV*_*S*_ is likely to exist in a super-Earth’s or an ocean planet’s solid core causing the locally seismic heterogeneity. Our results not only give some clues to explore and synthesize novel iron-rich iron oxides but also shed light on the fundamental information of oxygen in the planetary core.

## Introduction

Earth’s core is widely accepted to be dominantly composed of iron-nickel alloys. Light elements, which are cosmically abundant and soluble in iron at high pressures such as H, C, O, Si, S and P, are required to explain the 8~10% and 4~5% density deficit of Earth’s outer and inner core from pure iron, respectively^1–3^. Additionally, the positive density jump from Earth’s liquid outer core to the solid inner core at the inner-outer core boundary (ICB) is too large to be explained by the solidification across the ICB alone^4^. Therefore, both suggest that there should be more light elements in Earth’s liquid outer core.

Oxygen is thought to be an important candidate for compensating the density deficit of Earth’s core. Previous *ab initio* calculations and models have shown that oxygen strongly partitions into Earth’s liquid outer core from the solid inner core^5,6^. Therefore, much attention has been paid to explore oxygen in the outer core. For example, shockwave data in the Fe-S-O system demonstrated that adding oxygen into the liquid iron could not match the density and velocity profiles of the outer core simultaneously and indicated an oxygen-depleted outer core^7^. In contrast, *ab initio* molecular dynamic calculations found no oxygen-free iron alloy that fitted the seismological observations well^8^. This finding leads to the conclusion that oxygen is required as a major light element in Earth’s outer core and the best fitting result is 3.7 wt% O and 1.9 wt% Si without S or C in iron-nickel alloys^8^. Recent melting experiments have shown that O and Si could not exist at high concentrations simultaneously under outer core conditions and that SiO_2_ saturation sets limits on O and Si concentrations in Earth’s outer core^9^. These different results indicate that the amount of oxygen in the outer core remains elusive. On the other hand, investigations into the Fe-FeO system at high pressures give direct clues to determine the temperature at ICB and provide an alternative method to estimate the amount of oxygen in the outer core^10–12^. Melting experiments showed that the Fe-FeO system remains eutectic to at least 93 GPa^10^. However, the end member, FeO undergoes a series of transitions under higher pressure and temperature conditions, which will affect the phase relation of the Fe-FeO system^13–16^. This raises the question as to whether the Fe-FeO system keeps eutectic under core conditions.

In addition to oxygen-bearing iron alloys in Earth’s core, iron oxides have a widespread occurrence in Earth’s crust and mantle, which have a significant influence on the oxidation state and the phase balance of Earth’s interior. Therefore, extensive experiments have been conducted at relevant *P*-*T* conditions of Earth’s interior to investigate structural and physical properties of iron oxides^13,16–21^. In particular, recent studies have successfully synthesized a series of iron oxides with new stoichiometries at high *P*-*T* conditions such as Fe_4_O_5_, Fe_5_O_6_, Fe_5_O_7_, Fe_7_O_9_ and FeO_2_^21–26^. These findings have revealed a complex phase diagram of the Fe-O system at extreme conditions and indicated different scenarios in Earth’s interior. For example, Fe_4_O_5_ and Fe_5_O_6_ as well as those well-known iron oxides are stable and some of them are likely to coexist from 10 GPa to 20 GPa resulting in a complicated oxygen buffer in Earth’s transition zone^23,27,28^. However, it is obvious that these newly synthesized compounds are all oxygen-rich iron oxides with Fe/O < 1. Heretofore, investigations into iron-rich iron oxides with Fe/O > 1 are rare. Although some theoretical studies have been performed to explore iron-rich iron oxides under high pressure^29–32^, their structural and physical properties as well as thermodynamic stability with respect to other components in the Fe-O system are poorly understood.

Here we investigate the stability, elastic and seismic properties of (Fe_x_Ni_1−x_)_2_O under high pressure by first-principle calculations based on density functional theory (DFT). According to present results and previous data, we further discuss the existence of (Fe_x_Ni_1−x_)_2_O and its potential effect on geochemical and geophysical processes in Earth’s and a super-Earth’s interior.

## Results

**Figure 1:**
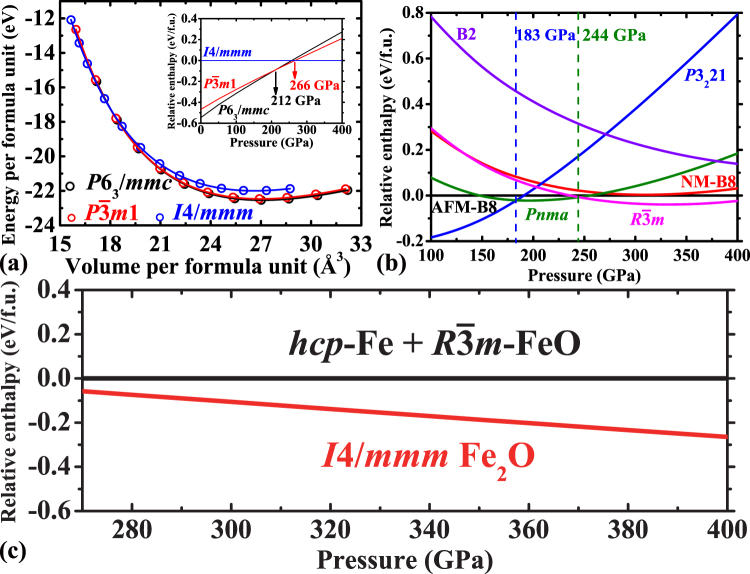
(**a**) The total energy vs. volume for Fe_2_O with *P*6_3_/*mmc* (black circles), $P\bar{3}m1$ (red circles) and *I*4/*mmm* (blue circles). The inset refers to calculated enthalpy differences as a function of pressure among the aforementioned structures. (**b**) Calculated enthalpy differences as a function of pressure for FeO with antiferromagnetic-B8 (black line), nonmagnetic-B8 (red line), *P*3_2_21 (blue line), *Pnma* (olive line), $R\bar{3}m$ (pink line) and B2 (purple line). (**c**) Calculated relative enthalpy of *I*4/*mmm*-Fe_2_O as a function of pressure compared with the assemblage of *hcp*-Fe and $R\bar{3}m$-FeO.

The static calculation results of Fe_2_O are displayed in Fig. 1(a) and the corresponding Equation of State (EoS) parameters are listed in Table S1. This figure illustrates that Fe_2_O undergoes structural transitions from *P*6_3_/*mmc* to $P\overline{3}m1$ at 212 GPa and further to *I*4/*mmm* at 266 GPa, consistent with previous simulations^32^. The later transition is accompanied by an increase of the coordination number of oxygen from six to eight. To investigate the dynamic stability of the *I*4/*mmm*-type Fe_2_O, *ab initio* lattice-dynamics calculations are performed [see Supplementary Fig. S2]. The phonon spectrum without any imaginary frequency implies that the *I*4/*mmm* structure is dynamically stable at 320 GPa.

In order to evaluate the relative stability of Fe_2_O versus FeO at pressures corresponding to Earth’s core, the following chemical reaction is considered in the present study:

1 $${\rm{FeO}}+{\rm{Fe}}\to {\rm{Fe2O}}$$

For FeO five candidate structures (*P*6_3_/*mmc* (B8), (B2), *P*3_2_21, *Pnma* and $R\overline{3}m$) are considered above 100 GPa in our simulations [see Supplementary Table S1]. Our simulations predict phase transitions form *P*3_2_21 to *Pnma* and further to $R\overline{3}m$ at 183 GPa and 244 GPa, respectively [Fig. 1(b)]. These results are inconsistent with previously experimental observations, where B8 is the most stable structure above 100 GPa at moderate temperatures and will transform into B2 above 240 GPa at high temperatures^13–15,33,34^. However, our results are in good agreement with previous theoretical calculations^32^. The present calculations are performed at 0 K (static calculations) while previous experiments were conducted at high temperatures. In addition, the ideal chemical stoichiometric compound FeO is considered in our simulations but the non-stoichiometric Fe_1−x_O was often used in experiments. For Fe the *hcp* structure is predicted to be stable at high pressures corresponding to Earth’s core compared with the *fcc* structure [see Supplementary Table S1]. Based on these parameters, we have calculated the relative enthalpy of *I*4/*mmm*-Fe_2_O as a function of pressure compared with the assemblage of *hcp*-Fe and $R\overline{3}m$-FeO [Fig. 1(c)]. Figure 1(c) demonstrates that *I*4/*mmm*-Fe_2_O becomes energetically favorable above ~270 GPa and is likely to be stable at least up to 400 GPa. Additionally, the enthalpy difference between *I*4/*mmm*-Fe_2_O and the assemblage of *hcp*-Fe and $R\overline{3}m$ $-$FeO increases with increasing pressure indicating that *I*4/*mmm*-Fe_2_O becomes more stable upon compression.

The compression of volumes as a function of pressure displayed in Fig. S3 illustrates a marginal volume change across the chemical reaction (1). The volume reduction of reaction (1) is about 1.45% at 260 GPa and increases with increasing pressure. Previous experiments have detected B2-FeO under ultra-high pressure and temperature conditions^15^ and therefore calculated results of the assemblage of *hcp*-Fe and B2-FeO are also included for comparison [see Supplementary Fig. S3]. Similarly, *I*4/*mmm*-Fe_2_O has a smaller volume compared with that of *hcp*-Fe + B2-FeO. Though the volume reduction of reaction (1) is marginal, generally smaller than 1.60% up to 400 GPa, it may have important contributions to stabilize the *I*4/*mmm*-type Fe_2_O at high pressures.

**Figure 2:**
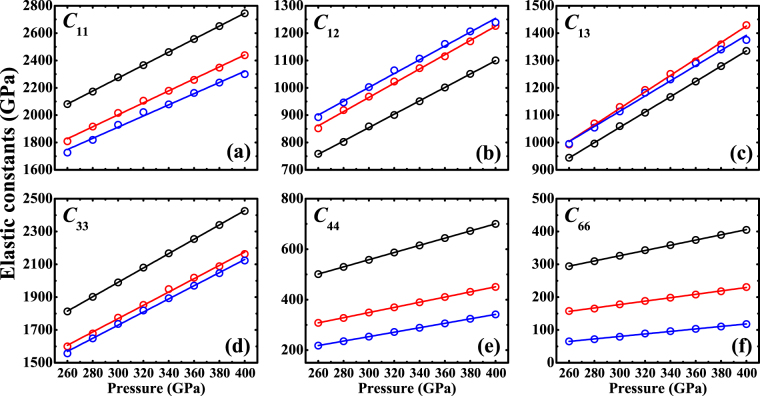
Calculated single-crystal elastic constants of *I*4/*mmm*-(Fe_x_Ni_1−x_)_2_O as a function of pressure. The black, red and blue open circles represent Fe_2_O, (Fe_0.5_Ni_0.5_)_2_O and Ni_2_O, respectively. The black, red and blue lines are obtained by linear fitting.

In order to evaluate the effect of Ni on elastic and seismic properties of Fe_2_O at high pressures, the components Ni_2_O and (Fe_0.5_Ni_0.5_)_2_O are also included in present simulations [see Supplementary Fig. S1(b) and Table S1]. (It is worth mentioning that different arrangements of Ni in (Fe_0.5_Ni_0.5_)_2_O were considered but they were unstable. When the atomic positions and unit-cell parameters were allowed to relax at each given volume, the *I*4/*mmm* structure would destroy.) The zero-pressure bulk modulus (*K*_0_) of (Fe_x_Ni_1−x_)_2_O decreases with the Ni content [see Supplementary Fig. S4]. The elastic constants of (Fe_x_Ni_1−x_)_2_O increase monotonically with pressure [Fig. 2 and see Supplementary Table S2]. These three components all present mechanically stable in the calculated pressure range supported by the Born-Huang criterion^35^. The substitution of Ni for Fe in (Fe_x_Ni_1−x_)_2_O tends to significantly decrease *C*_11_, *C*_33_, *C*_44_ and *C*_66_ and increase *C*_12_ and *C*_13_. Figure 2 also reveals a moderate anisotropy in the axial incompressibility along *a* axis and *c* axis. The *c* axis is more compressible than *a* axis with *C*_11_ > *C*_33_ for (Fe_x_Ni_1−x_)_2_O.

**Figure 3:**
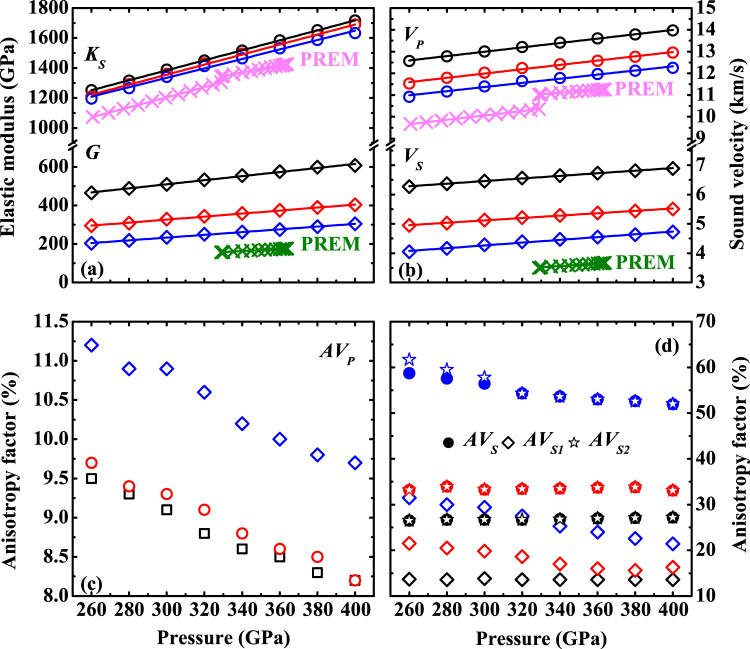
(**a**) Bulk and shear moduli (*K*_*S*_ and *G*) and (**b**) aggregate velocities (*V*_*P*_ and *V*_*S*_) of *I*4/*mmm*-(Fe_x_Ni_1−x_)_2_O as a function of pressure. (**c**) Variation of *P* wave anisotropies (*AV*_*P*_) and (**d**) shear wave splitting factors (*AV*_*S*_) and anisotropy factors of two polarized *S* waves (*AV*_*S1*_ and *AV*_*S2*_) for *I*4/*mmm*-(Fe_x_Ni_1−x_)_2_O at high pressures. The black, red and blue marks represent Fe_2_O, (Fe_0.5_Ni_0.5_)_2_O and Ni_2_O, respectively. The lines in (**a**) and (**b**) are obtained by linear fitting. The elastic and seismic profiles of PREM (pink and olive crosses) (ref ^37^) are plotted for comparison.

Using the aforementioned *C*_*ij*_ of (Fe_x_Ni_1−x_)_2_O, the adiabatic bulk and shear moduli (*K*_*S*_ and *G*) at high pressures are calculated according to the Voigt-Reuss-Hill averages^36^ [Fig. 3(a) and see Supplementary Table S2]. The *K*s and *G* of *I*4/*mmm*-(Fe_x_Ni_1−x_)_2_O increase monotonically with pressure and are all greater than PREM^37^. The substitution of Ni in (Fe_x_Ni_1−x_)_2_O slightly affects the *K*_*S*_ but significantly reduces the *G*. Furthermore, the aggregate *V*_*P*_ and *V*_*S*_ of (Fe_x_Ni_1−x_)_2_O are obtained from their moduli and densities [Fig. 3(b) and see Supplementary Table S2]. In contrast to the moduli of (Fe_x_Ni_1−x_)_2_O, the substitution of Ni in (Fe_x_Ni_1−x_)_2_O largely reduces *V*_*P*_ and *V*_*S*_ because both of them are partially controlled by the *G*.

To understand the evolution of velocity anisotropies of the *I*4/*mmm*-type (Fe_x_Ni_1−x_)_2_O as a function of pressure, the percentage of velocity anisotropies and their distributions are calculated based on *C*_*ij*_ and density at each given pressure^38,39^ [Fig. 3(c), (d) and see Supplementary Fig. S5, Table S2]. The *AV*_*P*_ of Fe_2_O is 9.5% at 260 GPa and decreases to 8.2% at 400 GPa [Fig. 3(c) and see Supplementary Table S3]. The *AV*_*P*_ of (Fe_0.5_Ni_0.5_)_2_O is similar to that of Fe_2_O while the *AV*_*P*_ of Ni_2_O is about 1.7% larger than that of Fe_2_O at each pressure point. These results indicate that the small substitution of Ni for Fe will not change the *AV*_*P*_ of (Fe_x_Ni_1−x_)_2_O significantly in the calculated pressure range. At 320 GPa, the fastest *P* wave (13.82 km/s) propagates along *a* axis direction and the slowest *P* wave (12.65 km/s) distributes in the (110) plane in Fe_2_O. The fastest and slowest *P* waves both reduce with the increasing amount of Ni in (Fe_x_Ni_1−x_)_2_O. It is worth mentioning that (Fe_x_Ni_1−x_)_2_O has a high *AV*_*S*_, i.e., the *AV*_*S*_ are 26.5%, 33.2% and 58.7% for Fe_2_O, (Fe_0.5_Ni_0.5_)_2_O and Ni_2_O at 260 GPa, respectively [Fig. 3(d)]. The high *AV*_*S*_ mainly results from the high anisotropy of *V*_*S2*_, though *AV*_*S1*_ is, in fact not small. The layered *I*4/*mmm* structure consists of OX_8_ blocks (X: Fe or Ni) along *c* axis contributing to a high *AV*_*S*_, which is similar to that of post-perovskite^40^.

## Discussion and Implications

Previous static simulations calculated the stability of ${Pm}\bar{3}m$-Fe_3_O and ${Pm}\bar{3}m$-Fe_4_O compared with the assemblage of *hcp*-Fe and B8-FeO under Earth’s core conditions^29^. The results have shown that the chemical combination of Fe and FeO into Fe_3_O or Fe_4_O is energetically unfavorable under Earth’s core pressures. Additionally, the enthalpy difference increases with pressure indicating the large instability of Fe_3_O or Fe_4_O even at higher pressures. A BiI_3_-like triclinic Fe_3_O was later found to possess much lower enthalpy than that of Fe_3_O with previously predicted structures^30^. But the enthalpy of this new phase was still over 1 eV higher than that of the assemblage of *hcp*-Fe and B8-FeO. The present results demonstrate that the *I*4/*mmm*-type Fe_2_O is energetically favorable than the *hcp*-Fe + $R\bar{3}m$-FeO assemblage. And the $R\bar{3}m$-FeO is more stable than B8-FeO based on our simulations. Thus, the *I*4*/mmm*-type Fe_2_O will be much more stable than the triclinic Fe_3_O at 0 K. Recent static calculations investigated the stability of $P\bar{6}m2$-Fe_3_O and *P*4/*nmm*-Fe_3_O at 350 GPa and 500 GPa, respectively by *ab initio* random structure searching^32^. These structures are still unstable with respect to the dissociation, though they are very close to the convex hull. Furthermore, the $P\bar{6}m2$ structure of Fe_3_O is found to be a mixture of phases consisting of Fe and Fe_2_O and the *P*4/*nmm* structure is found to consist of Fe, Fe_2_O and FeO. Therefore, combining previous data with our results, we propose that it is unlikely to form Fe_3_O or Fe_4_O by the chemical combination of Fe and Fe_2_O under Earth’s core pressures. Our static calculations indicate that the *I*4/*mmm*-type Fe_2_O is a stable phase in iron-rich iron oxides at 0 K.

The present static calculations have shown that the *I*4/*mmm*-type Fe_2_O is dynamically stable and has the smaller enthalpy and volume than those of the assemblage of Fe and FeO at high pressures, which all favor the formation of Fe_2_O by the combination of Fe and FeO. However, our high-pressure simulations are limited at 0 K in contrast to the high *P-T* conditions of Earth’s core. As shown in Fig. 1(c), the enthalpy difference between the *I*4/*mmm*-type Fe_2_O and the Fe + FeO assemblage at high pressure of the ICB is of the order of 0.15 eV corresponding to ~1750 K. In terms of B2-FeO, which has been experimentally confirmed at high *P-T* conditions, the enthalpy difference is of the order of 0.35 eV (~4100 K). Thus it is likely that the *I*4/*mmm*-type Fe_2_O can be stable at high *P-T* conditions. On the other hand, the entropy is going to play an important role at high temperature but its effect on the relative stability of the target system is not considered in the present study. Previous *ab initio* simulations have found that when the entropic effect is included in the calculation, the Gibbs free energy of the reaction, 62Fe + FeO → Fe_63_O, can be substantially reduced by ~3 eV^31^. As for the present target system, the introduction of entropic effect may correspondingly favor the combination of Fe and FeO at high *P-T* conditions. But, on the contrary, it can have an opposite effect causing the reaction energetically unfavorable. That means it might change our conclusion significantly and invalidate our further interpretations for the deep Earth at ultra-high temperature. Further investigations on the present target system by either *ab initio* molecular dynamics methods or high *P-T* experiments are definitely required to verify the reaction, Fe + FeO → Fe_2_O. It is to be noted that Earth’s core contains diverse light elements. The present of one light element can affect the relative stability and the amount of the other one in Earth’s core^8,9^. Thus, the relative stability and physical properties of the iron-rich iron oxide or oxygen-bearing iron alloys should be investigated as a function of not only pressure and temperature but also compositions in order to comprehensively elucidate the nature of oxygen in Earth’s core. But they are beyond the scope of the present study and we will work on them in the future.

In terms of phase relations of the Fe-FeO system, *Sherman* ^29^ indicated there should be no solid solution between Fe and FeO under Earth’s core conditions due to the aforementioned reasons. In contrast, later simulations implied that oxygen dissolved in iron might be stabilized at concentrations up to a few mol% under core conditions because of the significant entropic effect in the dilute solution^31^. Recent thermodynamic simulations suggested the feasibility of the ideal solution model to calculate the Fe-FeO liquid property under outer core conditions and yielded the eutectic compositions of Fe-7.2~9.1 wt% O^12^. Based on their thermodynamic simulations, they concluded that an overall oxygen-rich bulk outer core model should be excluded. In the present study, it is proposed that Fe_2_O can be a strong candidate phase for stable iron-rich iron oxides at high pressure, perhaps even at high temperature, which can potentially change phase relations of the Fe-FeO system at pressures corresponding to Earth’s lower outer core.

**Figure 4:**
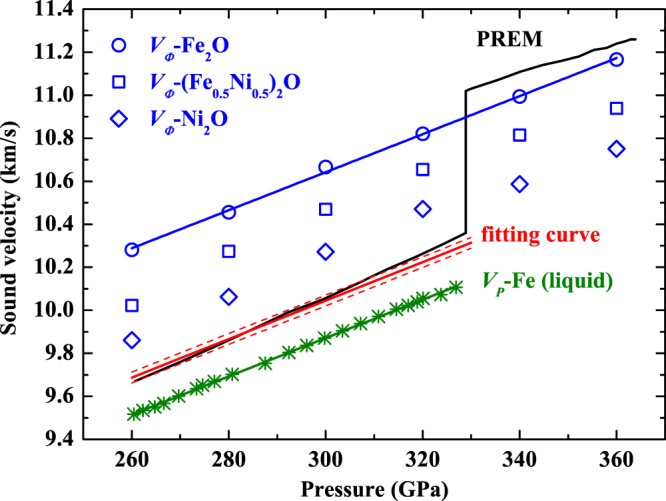
Bulk sound velocity of *I*4/*mmm*-(Fe_x_Ni_1−x_)_2_O (blue marks) as a function of pressure. The seismic profile of PREM (black line) (ref ^37^) and *V*_*P*_ profile of liquid iron (olivine stars) (ref ^42^) are plotted for comparison. The red fitting curve is calculated from 35 vol% *I*4/*mmm*-Fe_2_O + 65 vol% iron.

The accurate measurement of the sound velocity of liquid iron alloys at high pressure is very challenging. Previous studies have discussed the sound velocity of the liquid phase can be addressed from the bulk sound velocity of the solid phase with identical compositions^7^. In addition, theoretical calculations have shown that the bulk sound velocity (*V*_*φ*_) of the solid phase is comparable to *V*_*P*_ of the liquid phase (the difference is about 2.5%) and both are marginally dependent of temperature at a given high pressure^41,42^. Indeed, recent experiments have presumed *V*_*φ*_ of the solid FeS_2_ is equivalent to *V*_*P*_ of the liquid counterpart and thus estimated the sulfur content in Earth’s outer core^43^. Furthermore, in the *I*4/*mmm*-type Fe_2_O, the coordination number of oxygen is eight and each iron is surrounded by four oxygen and nine iron, which is similar to the recent report of structural properties of the liquid oxygen-bearing iron alloys by first-principles molecular dynamics^44^. Thus, it is reasonable that the target system can be used to roughly derive the amount of oxygen in the lower outer core. We obtain *V*_*φ*_ of (Fe_x_Ni_1−x_)_2_O as a function of pressure [Fig. 4 and see Supplementary Table S2] and estimate the volume fraction of Fe_2_O by the following relation:

2 $${V}_{P(PREM)}=\frac{0.975{V}_{\phi (solid-Fe2O)}{V}_{P(liquid-Fe)}}{(1-t)\cdot 0.975{V}_{\phi (solid-Fe2O)}+t{V}_{P(liquid-Fe)}}\,$$

where *t* is the volume fraction of Fe_2_O. We find a volume fraction, *t* ~ 35% [Fig. 4]. Then, if oxygen is the only light element in Earth’s lower outer core, the maximum possible oxygen content is ~4.3 wt%. However, thermoelastic parameters of (Fe_x_Ni_1−x_)_2_O at realistic outer core conditions are not well constrained, which certainly warrants further explorations.

On the other hand, for a terrestrial super-Earth, unlike Earth, the metal core is mainly composed of solid iron alloys and only a small portion of the core is liquid because of its high mass. Therefore, there will be more oxygen in a super-Earth’s solid core than that in Earth’s inner core assuming that they have similar cosmochemical compositions. There also exist a type of so-called ocean planets, where a thick layer of ice covers the rocky materials and the center temperature can be much lower than that of super-Earths’^45,46^. Based on current results at 0 K, above 270 GPa, it is energetically favorable for the formation of the *I*4/*mmm*-type Fe_2_O. The enthalpy difference becomes larger upon further compression, which can stabilize the *I*4/*mmm*-type Fe_2_O even at high temperature. Thus, it is likely that *I*4/*mmm*-(Fe_x_Ni_1−x_)_2_O could exist in the solid core of a super-Earth or an ocean planet. In addition, Fig. 3(d) demonstrates that *I*4/*mmm*-(Fe_x_Ni_1−x_)_2_O has a high *AV*_*S*_ in the calculated pressure range due to its layered structure. If (Fe_x_Ni_1−x_)_2_O can accumulate in the solid core locally, it will cause the locally seismic anisotropy in the solid core, which might be even observed by advanced instruments.

## Conclusions

In conclusion, the high-pressure behavior of (Fe_x_Ni_1−x_)_2_O has been studied based on first-principle density functional calculations. The end member Fe_2_O, is predicted to undergo structural transitions from *P*6_3_/*mmc* to $P\bar{3}m1$ at 212 GPa and further to *I*4/*mmm* at 266 GPa, consistent with previous simulations. The dynamically and mechanically stable *I*4/*mmm*-Fe_2_O becomes energetically favorable with respect to the assemblage of *hcp*-Fe and $R\bar{3}m$-FeO above 270 GPa. These results indicate that *I*4/*mmm*-Fe_2_O can be a strong candidate phase for stable iron-rich iron oxides at high pressure, perhaps even at high temperature, which requires further investigations by *ab initio* molecular dynamics methods or high *P-T* experiments. The elastic and seismic properties of *I*4/*mmm*-(Fe_x_Ni_1−x_)_2_O at high pressures have been discussed in detail. Combining previous data with present results, we roughly estimate that if oxygen is the only light element in Earth’s lower outer core, less than 4.3 wt% oxygen content is required to match the seismic observations. On the other hand, *I*4/*mmm*-(Fe_x_Ni_1−x_)_2_O may exist in a super-Earth’s or an ocean planet’s solid core. If (Fe_x_Ni_1−x_)_2_O can accumulate in its solid core locally, it is likely to cause the locally seismic heterogeneity because of its high *AV*_*S*_.

## Methods

Three candidate structures (*P*6_3_/*mmc*, $P\bar{3}m1$ and *I*4/*mmm* [see Supplementary Fig. S1(a)]) for Fe_2_O were considered in the present study and structural details could be found in Table S1. First-principle calculations were performed based on density functional theory with the projected augmented wave method (PAW) implemented in Vienna *ab-initio* simulation package (VASP)^47–49^. The Perdew-Burke-Ernzerhof (PBE) version of the generalized gradient approximations (GGA) was selected to treat the exchange correlation potential^50^. The kinetic energy cut-off was set to 1000 eV. The energy convergence criterion for the electronic self-consistent calculation was 10^−6^ eV. The total energy difference was converged to 1×10^−5^ eV/formula unit (f.u.) with respect to the energy cutoff and k-points, respectively. The force difference was converged to 1×10^−3^ eV/Å (less than 0.1 GPa). The spin-polarization of iron of Fe_2_O with various structures was not included because the existence of magnetic moments under Earth’s core conditions could be safely ruled out. For each crystalline phase, the atomic positions and unit-cell parameters were allowed to relax at each given volume to obtain the minimum total energy. Once the minimum total energies of each phase were obtained at different volumes, they were fitted to the third-order Birch-Murnaghan EoS^51,52^. Additionally, the enthalpy (*H* = *E* + *PV*) of each phase was compared with each other to identify the most stable structure at the given pressure.

The phonon dispersions were calculated using the Phonopy Code by the supercell method^53^ and a 2×2×2 supercell was constructed for the *I*4/*mmm*-type Fe_2_O. Single crystal elastic constants of *I*4/*mmm*-(Fe_x_Ni_1−x_)_2_O were computed from the stress-strain relations (*σ*_*ij*_ = *c*_*ijkl*_ *ε*_*kl*_, where *σ*_*ij*_ stands for the stresses, *c*_*ijkl*_ for the elastic moduli and *ε*_*kl*_ for the strains)^54^. For the *I*4/*mmm* symmetry, we derived six independent elastic constants (*C*_11_, *C*_12_, *C*_13_, *C*_33_, *C*_44_ and *C*_66_). We applied positive and negative strains of magnitude of 0.5% in order to accurately determine the stresses in the appropriate limit of zero strain.

## Electronic supplementary material

Supplementary Material
