# Supplementary material for: Stability and anisotropy of (FexNi1−x)2O under high pressure and implications in Earth’s and super-Earths’ core
Source: Sci Rep. 2018 Jan 10;8:236. doi: 10.1038/s41598-017-18678-z (PMC5762755; doi:10.1038/s41598-017-18678-z)
Supplement: Supplementary file 1 — Supplementary Material [file 41598_2017_18678_MOESM1_ESM.pdf]

Supplementary Material

Stability and anisotropy of $(Fe_xNi_{1-x})_2O$ under high pressure and implications in Earth's and super-Earth's core

Shengxuan Huang[1,2], Xiang Wu[1,*], and Shan Qin[2]

[1] *State key laboratory of geological processes and mineral resources, China University of Geosciences (Wuhan), 430074, P. R. China.*

[2] *Key Laboratory of Orogenic Belts and Crustal Evolution, MOE, Peking University and School of Earth and Space Sciences, Peking University, Beijing 100871, P. R. China.*

[*] Corresponding author: wuxiang@cug.edu.cn

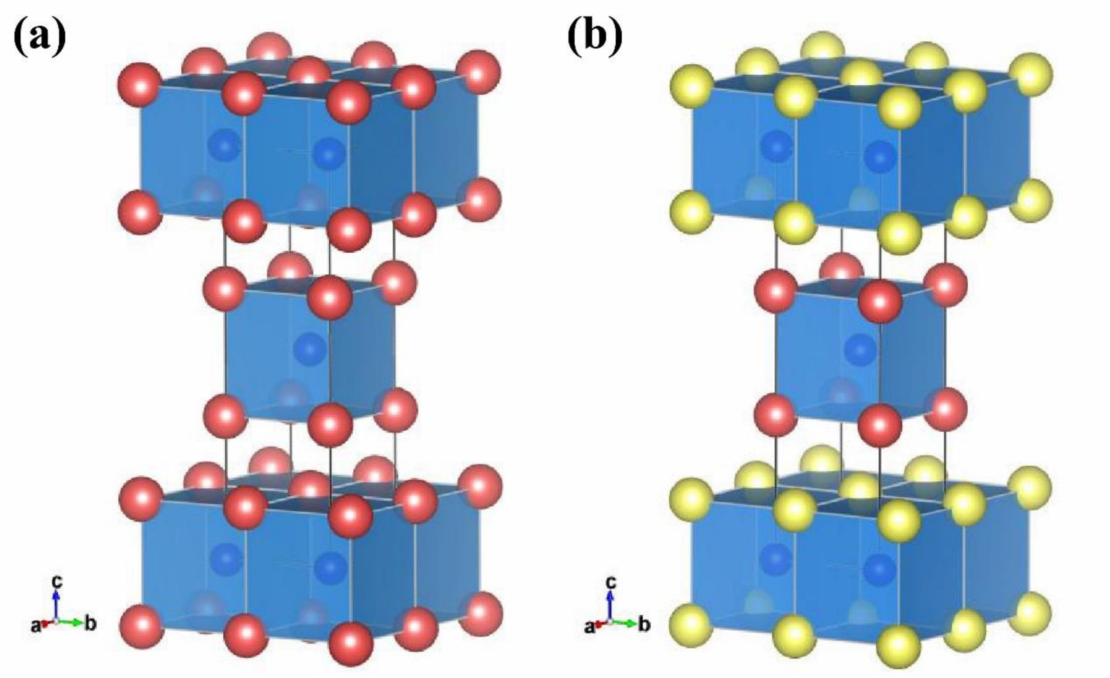

Fig. S1. The *I*4/*mmm*-type structure of (a) Fe_2O and (b) $(Fe_{0.5}Ni_{0.5})_2O$. The blue, red and yellow objects represent O anion, Fe cation and Ni cation, respectively.

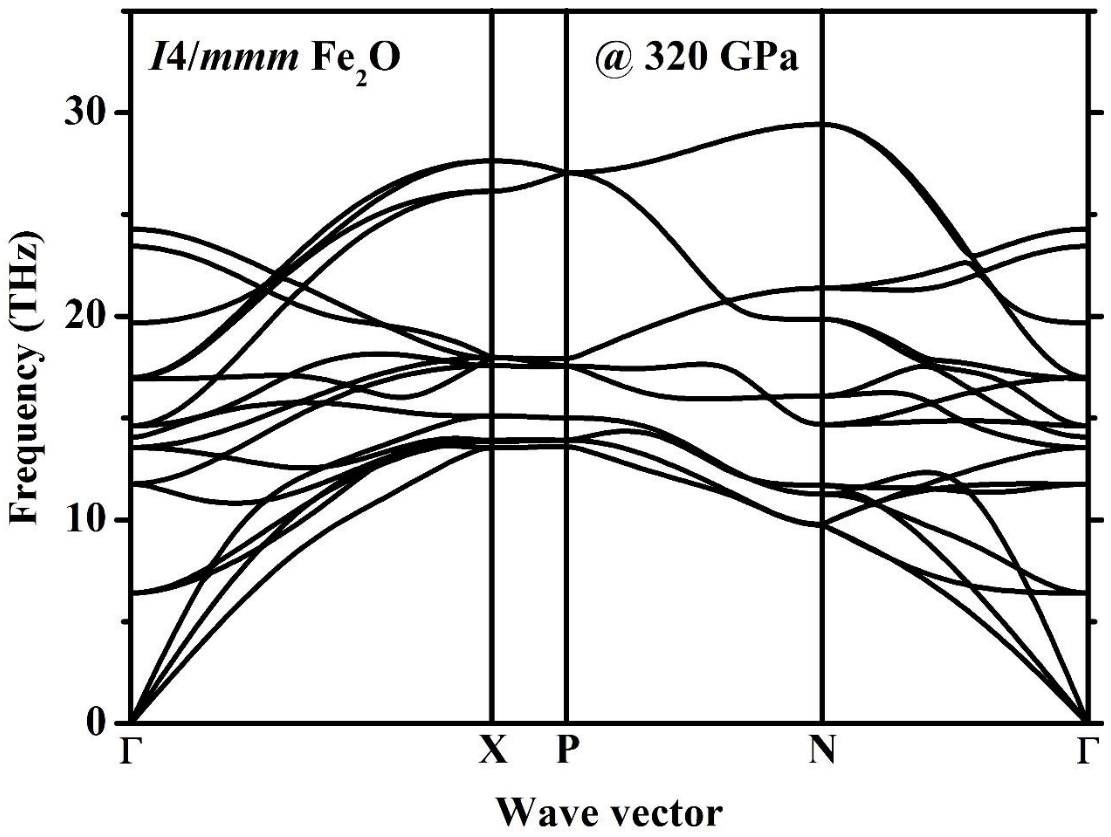

Fig. S2. The phonon dispersion of the *I*4/*mmm*-type Fe_2O at 320 GPa.

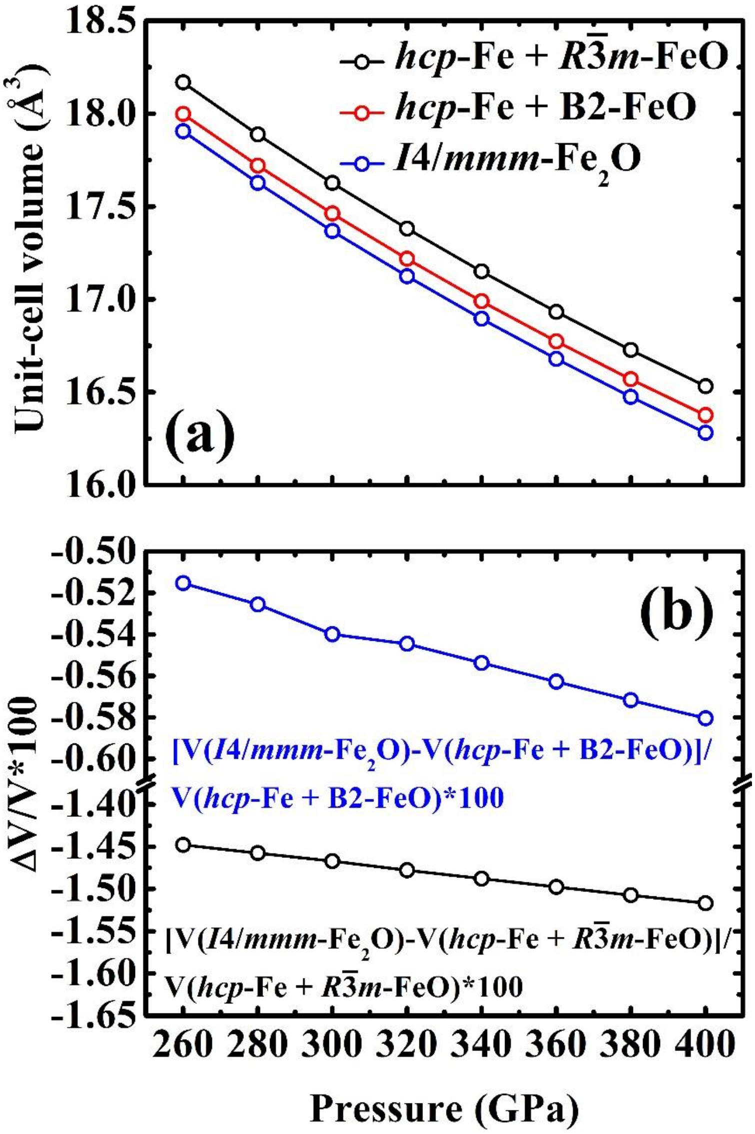

Fig. S3. (a) Unit-cell volume of different phases as a function of pressure. (b) The volume differences (ΔV) relative to that of the assemblage of Fe and FeO at various pressures.

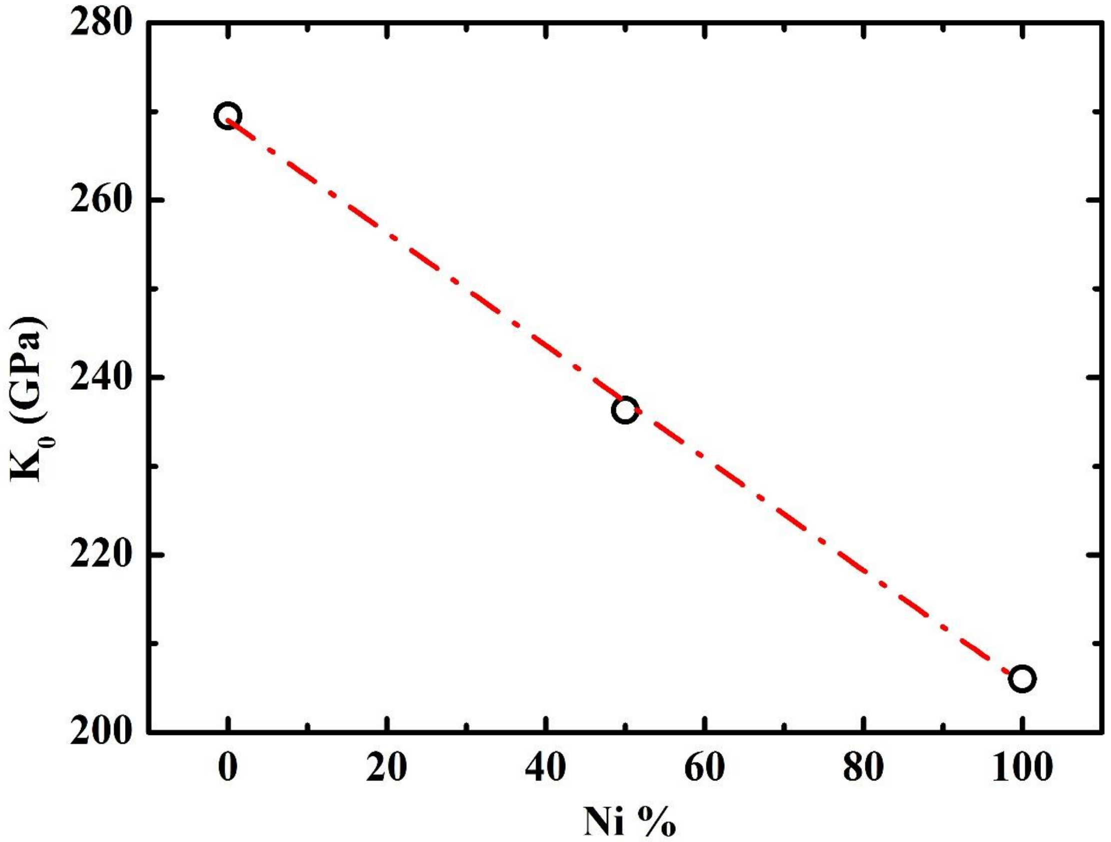

Fig. S4. The zero-pressure bulk modulus of the *I*4/*mmm*-$(Fe_xNi_{1-x})_2O$ as a function of Ni content. The red dashed line is obtained by the linear fitting.

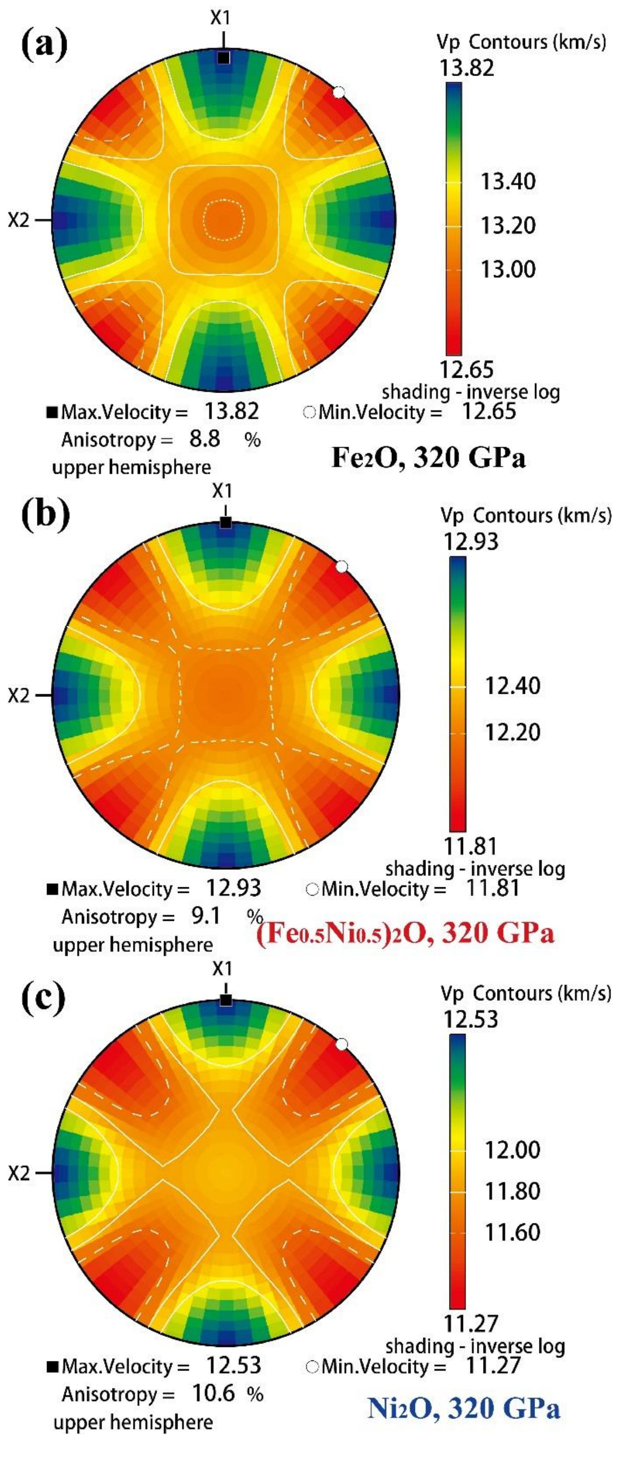

Fig. S5. *P* wave velocity distributions for the *I*4/*mmm*-$(Fe_xNi_{1-x})_2O$ at 320 GPa. All pole figures are the upper hemisphere projections.

Table S1 Calculated parameters of the third-order Birch-Murnaghan equation of state (energy per formula unit E_0, volume per formula unit V_0, bulk modulus K_0, its pressure derivative K_0' at zero pressure) of different phases. AFM and NM represent antiferromagnetic and non-magnetic, respectively.

Phase	Formula units	E_0/f.u. (eV)	V_0 /f.u. (Å^3)	K_0 (GPa)	K_0'
			Fe_2O		
$P6_3/mmc$	2	-22.54	27.07	265.0	4.52
$P\bar{3}m1$	1	-22.46	26.98	267.4	4.49
$I4/mmm$	2	-22.00	26.63	269.5	4.42
			Fe		
$Im\bar{3}m$	2	-7.98	10.56	268.3	4.47
$P6_3/mmc$	2	-8.37	10.26	289.4	4.46
			FeO		
AFM-B8 ($P6_3/mmc$)	2	-15.20	19.62	138.1	4.32
NM-B8 ($P6_3/mmc$)	2	-14.14	16.95	242.1	4.51
B2 ($Pm\bar{3}m$)	1	-13.44	16.50	253.4	4.48
$P3_221$	3	-15.06	17.64	263.3	4.48
$Pnma$	4	-14.46	17.10	251.0	4.52
$R\bar{3}m$	3	-14.08	16.82	247.1	4.52
			$(Fe_{0.5}Ni_{0.5})_2O$		
$I4/mmm$	2	-18.97	27.47	236.3	4.47
			Ni_2O		
$I4/mmm$	2	-16.40	28.25	206.0	4.51

Table S2 Single-crystal and aggregate elastic properties of the *I*4/*mmm*-type $(Fe_xNi_{1-x})_2O$ at high pressures.

**Pressure (GPa)**	C_{11} **(GPa)**	C_{12} **(GPa)**	C_{13} **(GPa)**	C_{33} **(GPa)**	C_{44} **(GPa)**	C_{66} **(GPa)**	K_S **(GPa)**	***G*** **(GPa)**	V_P **(km/s)**	V_S **(km/s)**	V_φ **(km/s)**
					*I*4/*mmm* Fe_2O						
260	2081	759	945	1813	501	294	1252	465	12.57	6.27	10.28
280	2173	802	996	1902	530	310	1315	488	12.78	6.37	10.46
300	2277	859	1060	1989	558	326	1389	509	13.01	6.46	10.67
320	2366	901	1109	2080	587	343	1450	533	13.21	6.56	10.82
340	2462	951	1166	2168	615	359	1517	555	13.41	6.65	10.99
360	2557	1001	1223	2255	644	374	1585	576	13.61	6.73	11.17
380	2652	1051	1280	2340	672	390	1652	598	13.79	6.81	11.33
400	2745	1100	1335	2425	700	405	1717	608	13.97	6.89	11.48
					*I*4/*mmm* $(Fe_{0.5}Ni_{0.5})_2O$						
260	1809	852	993	1600	308	158	1210	295	11.53	4.95	10.02
280	1917	918	1070	1679	328	165	1292	308	11.79	5.02	10.27
300	2016	968	1130	1775	349	178	1362	328	12.03	5.13	10.47
320	2106	1023	1192	1853	370	188	1431	343	12.24	5.21	10.65
340	2178	1072	1251	1949	390	198	1495	358	12.42	5.29	10.82
360	2258	1115	1297	2019	411	208	1550	374	12.58	5.37	10.94
380	2348	1170	1359	2089	431	218	1618	389	12.76	5.44	11.11
400	2439	1226	1429	2163	450	231	1690	403	12.95	5.51	11.28
					*I*4/*mmm* Ni_2O						
260	1726	892	995	1557	218	65	1196	203	10.92	4.05	9.86
280	1819	947	1054	1649	235	72	1266	218	11.16	4.16	10.06
300	1930	1003	1114	1737	253	79	1340	234	11.40	4.28	10.27

320	2022	1064	1182	1818	272	89	1413	250	11.64	4.39	10.47
340	2079	1107	1231	1894	289	96	1465	262	11.78	4.47	10.59
360	2163	1161	1290	1970	306	103	1531	275	11.97	4.55	10.75
380	2238	1206	1340	2046	324	110	1588	289	12.12	4.63	10.88
400	2300	1239	1375	2124	341	118	1633	304	12.25	4.72	10.96

Table S3 Seismic anisotropies of *P*- and two polarized S-waves (AV_P, AV_{S1} and AV_{S2}) and shear wave splitting factor (AV_S) of the *I*4/*mmm*-type $(Fe_xNi_{1-x})_2O$ at high pressures.

Pressure (GPa)	AV_P (%)	AV_S (%)	AV_{S1} (%)	AV_{S2} (%)
		*I*4/*mmm* Fe_2O		
260	9.5	26.5	13.7	26.5
280	9.3	26.7	13.6	26.7
300	9.1	26.7	13.8	26.7
320	8.8	26.7	13.6	26.7
340	8.6	26.8	13.6	26.8
360	8.5	27.0	13.6	27.0
380	8.3	27.1	13.6	27.1
400	8.2	27.2	13.6	27.2
		*I*4/*mmm* $(Fe_{0.5}Ni_{0.5})_2O$		
260	9.7	33.2	21.5	33.2
280	9.4	33.9	20.5	33.9
300	9.3	33.3	19.8	33.3
320	9.1	33.4	18.6	33.4
340	8.8	33.5	17.0	33.5
360	8.6	33.7	16.0	33.7
380	8.5	33.8	15.6	33.8
400	8.2	33.1	16.3	33.1
		*I*4/*mmm* Ni_2O		
260	11.2	58.7	31.5	61.7
280	10.9	57.6	30.0	59.5
300	10.9	56.5	29.4	57.8
320	10.6	54.3	27.5	54.3
340	10.2	53.6	25.3	53.6
360	10.0	53.0	24.0	53.0
380	9.8	52.6	22.6	52.6
400	9.7	52.0	21.4	52.0
